# Moral “foundations” as the product of motivated social cognition: Empathy and other psychological underpinnings of ideological divergence in “individualizing” and “binding” concerns

**DOI:** 10.1371/journal.pone.0241144

**Published:** 2020-11-10

**Authors:** Michael Strupp-Levitsky, Sharareh Noorbaloochi, Andrew Shipley, John T. Jost

**Affiliations:** 1 Department of Psychology, Long Island University-Brooklyn, New York, NY, United States of America; 2 Goldman Sachs, New York, NY, United States of America; 3 AGS Law, Palo Alto, California, United States of America; 4 Departments of Psychology, Politics, and Data Science, New York University, New York, NY, United States of America; Saint Peter's University, UNITED STATES

## Abstract

According to moral foundations theory, there are five distinct sources of moral intuition on which political liberals and conservatives differ. The present research program seeks to contextualize this taxonomy within the broader research literature on political ideology as motivated social cognition, including the observation that conservative judgments often serve system-justifying functions. In two studies, a combination of regression and path modeling techniques were used to explore the motivational underpinnings of ideological differences in moral intuitions. Consistent with our integrative model, the “binding” foundations (in-group loyalty, respect for authority, and purity) were associated with epistemic and existential needs to reduce uncertainty and threat and system justification tendencies, whereas the so-called “individualizing” foundations (fairness and avoidance of harm) were generally unrelated to epistemic and existential motives and were instead linked to empathic motivation. Taken as a whole, these results are consistent with the position taken by Hatemi, Crabtree, and Smith that moral “foundations” are themselves the product of motivated social cognition.

## Introduction

“There is a wide and varied range of motivations: substantive and formal, conscious and unconscious, self-regarding and non-self-regarding, forward-looking and backward-looking.” (Jon Elster, [1])

One of the earliest and, as it turns out, most durable insights of social and political psychology is that people hold the beliefs, opinions, and values they do for reasons that are far from self-evident—both to themselves and to others. More than a century ago, for instance, Graham Wallas, a Fabian socialist and co-founder of the London School of Economics, argued against the “intellectualist” assumption that political judgment is driven largely by “calculations of means and ends” in a 1908 work entitled *Human Nature in Politics* [2]. Wallas was especially worried that democracies were vulnerable to elite manipulation of the masses through “the creation of opinion by the deliberate exploitation of subconscious non-rational inference.” Tragically, the course of European history in the 20^th^ century proved that his worries were well-founded [3–5].

## Theory of ideology as motivated social cognition

In many ways, the serious psychological investigation of political ideology began with Adorno, Frenkel-Brunswik, Levinson, and Sanford‘s [6] classic volume on *The Authoritarian Personality*. The book has been much maligned on both methodological and ideological grounds, but the fact remains that it represented a profound synthesis of social, personality, and political psychology that has much to offer contemporary readers [7,8]. In this work, Adorno and colleagues formulated an indispensable theoretical assumption in psychology, namely that specific “ideologies have for different individuals, different degrees of appeal, a matter that depends upon the individual’s needs and the degree to which these needs are being satisfied or frustrated” [6, p. 2].

The same assumption was made by Smith, Bruner, and White [9], who put forth a “functional” theory of attitudes in *Opinions and Personality* [see also 10,11]. The guiding insight was that individuals hold the beliefs, opinions, and values they do because they address underlying psychological needs or interests, such as those related to self-esteem maintenance, group cohesion, or rationalization of the social order [12]. Political ideologies, in other words, serve social and psychological functions (or motives) that may or may not be entirely rational—or motivated by objective self-interest—but nevertheless help to explain why people are drawn to them in the first place.

It was in this spirit that Jost, Glaser, Kruglanski, and Sulloway [13,14] proposed a theory of political ideology as motivated social cognition. The basic notion was that people whose psychological needs to reduce uncertainty and threat were either chronically or temporarily activated would be attracted to conservative (or rightist) ideology and repulsed by liberal (or leftist) ideology. To understand why this would be so, it is useful to draw on the concept of *system justification*, which is defined as the (conscious or nonconscious) motivation to defend, bolster, and justify existing social, economic, and political arrangements [15]. Although nearly everyone is motivated to justify the status quo to at least some degree, conservatives consistently score higher than liberals on epistemic and existential needs to reduce uncertainty and threat as well as measures of general or “diffuse” system justification. If one is highly motivated to attain a subjective sense of certainty, predictability, control, safety, security, and reassurance, it stands to reason that the legitimation and preservation of what is familiar and comforting—the traditional status quo—would be more appealing than the open-ended struggle for a new and better social system.

Although there is by now a great deal of evidence to back up the theory of political conservatism as motivated social cognition [e.g., 16–18], the framework was attacked early on by conservative pundits who failed to appreciate a psychological analysis of their underlying motives. Ann Coulter, for instance, accused the four authors of “treason”:

The study also explained that “conservatives don’t feel the need to jump through complex, intellectual hoops in order to understand or justify some of their positions… “Whenever you have backed a liberal into a corner—if he doesn’t start crying—he says, “It’s a complicated issue.” Loving America is too simple an emotion. To be nuanced you have to hate it a little. Conservatives may not grasp “nuance,” but we’re pretty good at grasping treason.

Another right-wing pundit, Jonah Goldberg, fumed about the Jost et al. [13] article in the *Wall Street Journal* before waxing nostalgic for a bygone era of police crackdowns on campus radicals:

Now that I’ve calmed down a bit (read: I’ve put the shotgun down, and put my car keys back on the table), I thought I’d include a “relaxation aid” at the end of this post to help calm down people who read that whole thing. Here’s a photo from when Governor Ronald Reagan ordered a tear gas airstrike on UC Berkeley’s Sproul Plaza during an anti-war demonstration…. The best part is that we don’t have to pine for an “idealized past.” In the real past, lefty nutjobs at Berkeley really were heavily sprayed with teargas.

To astute observers, the defensive hostility and aggressive, authoritarian posturing expressed in these outbursts did more to confirm than to disconfirm the tenets of the theory of political conservatism as motivated social cognition [e.g., 19].

The theory of ideology as motivated social cognition was criticized by a few prominent social scientists as well. Mitchell and Tetlock [20], for instance, questioned the very enterprise of investigating “subterranean motives” for political ideologies. But, as noted above, there is a long and illustrious intellectual tradition that can be traced back to Graham Wallas and Harold Lasswell, the founders of political psychology, that analyzes belief systems in terms of their motivational (or functional) bases. Indeed, Elster [1] argued that motivational analyses are essential to the study of political psychology and that they have long been central to important historical and philosophical contributions by the likes of Alexis de Tocqueville and John Stuart Mill.

## “Moral foundations” as an alternative to theories of motivated social cognition

Probably the harshest criticism of the theory of ideology as motivated social cognition within the discipline came from proponents of “moral foundations theory.” Haidt and Graham [21, p. 101] took issue with the claim made by Jost et al. [13, p. 340] that “people embrace conservatism in part ‘because it serves to reduce fear, anxiety, and uncertainty; to avoid change, disruption, and ambiguity, and to explain, order, and justify inequality among groups and individuals.” According to Haidt and Graham, such a view “leads to open expressions of self-righteousness and contempt” and devalues conservative moral principles “that liberals do not acknowledge to be moral principles, such as unconditional loyalty to one’s group, respect for one’s superiors, and the avoidance of carnal pleasures” [13, p. 101]. These authors claimed that a more ideologically “heterodox” account of political psychology would recognize that liberals have an impoverished sense of morality, because they emphasize only issues of fairness and the avoidance of harm (the so-called “individualizing foundations”), whereas conservatives have a broader “moral palette” that values ingroup loyalty, obedience to authority, and the enforcement of purity sanctions (i.e., the “binding foundations”; see also [22]).

Moral foundations theorists make much of the fact that conservatives value ingroup, authority, and purity significantly more than liberals do, but they consistently overlook the fact that *liberals value fairness and the avoidance of harm significantly more than conservatives do* [21,22]. Kugler, Jost, and Noorbaloochi [23] observed not only that self-identified conservatism was positively and significantly correlated with the endorsement of “binding foundations,” but in all cases it was also *negatively* and *significantly* correlated with the endorsement of “individualizing foundations” (see their Table 1). To our knowledge, no explanation for why conservatives would express significantly *less* concern than liberals about fairness and harm avoidance has been offered by moral foundations theorists. Indeed, this finding might be taken to suggest that conservatives have a less—rather than more—discerning “moral palette”.

**Table 1:** Descriptive statistics including intercorrelations of study variables (Study 1, *N* = 225 NYU students).

		*M*	*SD*	1	2	3	4	5	6	7	8	9	10	11	12
**Motivation Variables**	(1) Basic Empathy	3.86	0.38	-											
	(2) Need for Closure	3.71	0.45	.06	-										
	(3) Need for Cognition	3.32	0.60	.15*	-.39**	-									
	(4) Death Anxiety	4.84	1.64	.04	.20*	-.10	-								
**Moral Intuition Variables**	(5) Avoidance of Harm	3.55	0.79	.40**	.09	.09	.01	-							
	(6) Fairness Concerns	3.76	0.69	.28**	.10	.06	.03	.58**	-						
	(7) Ingroup Loyalty	2.81	0.78	.12	.24**	-.22**	.19**	.12	.14*	-					
	(8) Obedience to Authority	2.73	0.81	-.03	.33**	-.32**	.17**	.06	.14*	.56**	-				
	(9) Purity Concerns	2.79	0.99	.08	.30**	-.26**	.31**	.13*	.14*	.48**	.58**	-			
	(10) Humanizing Concerns	3.66	0.66	.38**	.11	.09	.02	.90**	.87**	.15*	.11	.15*	-		
	(11) Binding Concerns	2.78	0.72	.07	.35**	-.32**	.28**	.13	.16*	-.80**	.85**	.85**	.16*	-	
**Ideology**	(12) Economic SJ	4.84	0.90	-.12	.20**	-.26**	.14*	-.19**	-.23**	.40**	.36**	.31**	-.23**	.43**	-
	(13) Liberalism-Conservatism	4.72	1.79	-.08	.19**	-.22**	.06	-.15*	-.10	-.35**	.28**	.30**	-.15*	.37**	.44**

Note. SJ = Systemic Justification

**. Correlation is significant at the .01 level (2-tailed).

*. Correlation is significant at the .05 level (2-tailed).

An important plank in Haidt and Graham’s [21] argument is that psychological differences between liberals and conservatives are attributable to differences in *genuinely* moral intuitions (or “foundations”) *rather than*, say, epistemic or existential motives, which are fundamentally non-moral motivations. This, plank, in turn, is used to buttress Haidt’s [22] normative argument that liberals *should value* conservative preferences for ingroup loyalty, obedience to authority, and the enforcement of purity sanctions—or at least *tolerate* them, which amounts to the same thing, namely valuing them positively rather than negatively or not at all. Unfortunately, there are a number of serious conceptual and methodological problems with moral foundations theory and the empirical grounds on which it rests [23–35].

For the purposes of the present article, an important criticism is that Haidt and Graham [21] provide no psychological evidence to suggest that the “binding foundations”—ingroup loyalty, obedience to authority, and the enforcement of purity sanctions—are motivated by ethical concerns in anything like the same sense that fairness and harm avoidance are [23,25,27,32,33]. Moral foundations theorists simply point out that conservatives—like former U.S. Senator Rick Santorum—*believe* that their opinions are based on fundamentally *moral* concerns. Haidt and Graham (21, p. 111) explain, for instance, that “Santorum’s anti-gay-marriage views were based on concerns for traditional family structures, Biblical authority, and moral disgust for homosexual acts (which he had previously likened to incest and bestiality).” The fact that Santorum’s concerns—like those of Ann Coulter and Jonah Goldberg (reproduced above)—appear to have much more in common with right-wing authoritarianism, social dominance orientation, and system justification motivation than with any sophisticated form of moral reasoning is either overlooked or sidestepped by moral foundations theorists.

To date, researchers inspired by moral foundations theory have provided no evidence bearing on the actual moral standing of conservative preferences for ingroup loyalty, deference to authority, and the enforcement of purity sanctions. The have instead relied upon the fallacious rhetorical technique of “argument by assertion,” simply declaring that such preferences are “moral (instead of amoral, or immoral)” rather than providing any evidence for such a declaration (21, p. 113). When other researchers have administered tests of moral sophistication in the tradition of Piaget and Kohlberg alongside the moral foundations questionnaire, they have observed that the endorsement of “binding foundations” is associated with conventional forms of moral reasoning that simply emphasize the maintenance of existing social norms and the established social order [24,27].

This evidence is broadly consistent with a plethora of recent studies demonstrating that endorsement of the “binding foundations” is positively associated with authoritarianism, social dominance orientation, outgroup prejudice, and victim-blaming in cases of rape and other violent crimes [23,35–46]. Thus, there is no evidence that prioritizing ingroup loyalty, deference to authority, and the enforcement of purity sanctions is associated with genuinely moral reasoning and plenty of evidence to suggest the opposite. Endorsement of the “individualizing foundations”—fairness and harm avoidance—on the other hand, is *negatively* associated with these same antisocial tendencies—as one would expect from a “moral” motivation. For instance, people who endorse the “individualizing foundations” are significantly more likely to donate to various charities and report online hate speech, whereas people who endorse the “binding foundations” are significantly less likely to do these things [47,48]. Furthermore, people who endorse the “binding foundations” are more likely to condone unethical business activities, whereas people who endorse the “individualizing foundations” are more likely to condemn them [49].

There is also growing evidence that liberals are more strongly motivated than conservatives by empathic concerns for a wider circle of people [50–55]—as well as the ethical treatment of nonhuman animals [56–58]. Thus, there is reason to suspect that liberals’ and conservatives’ moral intuitions (or judgments) are motivated by psychological factors, but that these psychological factors are not the same [13,14,59,60]. As Elster [1] pointed out, some motives are “self-regarding” while others are “non-self-regarding,” some are “forward-looking,” and others are “backward-looking.” In the present research program, we explore the possibility that those moral foundations that are more appealing to liberals than conservatives—namely, fairness and harm avoidance—are linked to empathic motivation, whereas the moral foundations that are more appealing to conservatives than liberals—ingroup loyalty, deference to authority, and the enforcement of purity sanctions—are not.

Another problem is that Haidt and Graham’s (21) juxtaposition of theories of political conservatism as motivated social cognition and moral foundations relies upon a false dichotomy. As noted above, they argue that conservatives are motivated—*not by* epistemic or existential concerns or system justification tendencies—but by purely “moral (instead of amoral, or immoral)” concerns (21, p. 113). Thus, they do not even consider the possibility that *moral preferences favored by conservatives—including preferences for ingroup loyalty*, *deference to authority*, *and the enforcement of purity sanctions—are themselves linked to psychological needs and motives to reduce uncertainty and threat and to rationalize the legitimacy of the status quo*. This is the possibility that we investigate in the present research program, which seeks to integrate theories of moral foundations and motivated social cognition rather than setting up a false juxtaposition between them. In other words, “moral” intuitions about fairness, harm, loyalty, authority, and purity—like the broader political ideologies in which they are embedded—may be related to the same sets of underlying psychological needs and motives identified in other research programs.

Recent work by Hatemi et al. [29] lends credence to the notion that moral intuitions are themselves the product of ideological justifications or rationalizations—rather than the origins of ideological differences, as Haidt and Graham (21) claim. For one thing, Hatemi and colleagues find that the test-retest reliability of the moral foundations questionnaire is much lower than the test-retest reliability of symbolic and operational measures of ideology. This is inconsistent with the notion that “moral foundations” are evolutionarily derived, genetically stable predispositions that explain the origins of political orientation, as moral foundations theorists have long claimed. Second, Hatemi and colleagues analyze longitudinal data and demonstrate that political ideology is a much stronger predictor of subsequent moral intuitions than vice versa. The authors conclude that these results are at odds with the assumptions of moral foundations theory and that they “support theoretical frameworks suggesting moral evaluations are more rooted in motivated reasoning anchored in political beliefs” (p. 16), as in the case of Jost et al.’s [13,14] theory of ideology as motivated social cognition.

There is also evidence that feelings of threat are associated with endorsement of “binding” (but not “individualizing”) moral foundations. For instance, Van Leeuween and Park [45] observed that individuals who scored relatively high on “perceptions of dangerous world” were more likely to prioritize ingroup loyalty, obedience to authority, and the enforcement of purity sanctions. Two other studies suggest that temporarily heightening existential motivation increases the psychological appeal of “binding” moral foundations. Van de Vyver et al. [43] observed in two nationally representative samples that survey respondents were more enthusiastic about ingroup loyalty and less enthusiastic about fairness shortly after (vs. shortly before) the London bombings of July 7, 2005. These respondents also exhibited greater prejudice toward Muslims and immigrants after the attack. Furthermore, Tamborini, Hofer, Prabhu, and Klebig [61] demonstrated that exposure to news about a terrorist attack increased the salience of obedience to authority and, relatedly, undermined prosocial behavioral intentions with respect to outgroup members. The results of these studies suggest that theories of moral foundations and motivated social cognition are in fact compatible rather than incompatible, as noted by Van Leeuween and Park [45].

## Overview of the present research program

In contrast to Haidt and Graham (21), we believe that incorporating (rather than setting aside) the theory of political ideology as motivated social cognition—and especially the idea that conservative attitudes serve system-justifying functions of maintaining the existing social order—will help to illuminate the vicissitudes of moral judgment, much as it has illuminated the vicissitudes of political judgment [13,14,17,59]. More specifically, we hypothesized that—far from being irrelevant to “binding” moral intuitions—epistemic and existential needs to reduce uncertainty and threat would be associated with “values” (or “vices”) that appeal to people who are relatively conservative and high in authoritarianism and social dominance, namely preferences for ingroup loyalty, deference to authority, and the enforcement of purity sanctions [see also 23,45].

Conversely, we expected that the kinds of values that appeal to people who are relatively liberal and low in authoritarianism and social dominance, namely preferences for fairness and the avoidance of harm, would be associated with empathic forms of motivation. This hypothesis is consistent with the frequent observation that liberals exhibit more empathic concern toward a wider variety of social targets, in comparison with conservatives [e.g., 50–55]. Thus, we explored the possibility that empathy would be positively associated with the endorsement of “individualizing” (but not “binding”) moral foundations, further underscoring the notion that moral intuitions such as fairness and the avoidance of harm that are especially appealing to liberals are qualitatively different than the more authoritarian and system-justifying moral intuitions that appeal to conservatives.

We also supposed that system justification processes would not be irrelevant to moral intuitions, as Haidt and Graham (21) implied, and that they would instead help to address the question of *why* specific moral intuitions are linked to political ideology [see 29]. Specifically, we hypothesized that the endorsement of “binding foundations” would be positively associated with system justification—consistent with the notion that this type of moral reasoning is conventional and preoccupied with the maintenance of existing social norms and the established social order [27]. The endorsement of “individualizing foundations,” on the other hand, should be negatively associated with system justification, insofar as pushing for fairness and the reduction of harm directly challenges institutionalized injustice and exploitation in society [15].

In the present research program, we therefore used a combination of regression and path modeling techniques to test an integrative model that incorporates epistemic and existential motives as well as “binding” and “individualizing” moral intuitions in order to provide a more complete and accurate picture of the motivational underpinnings of moral judgment and political ideology. Although a previous research program by Kugler et al. [23] provided some evidence that the endorsement of “binding” moral intuitions was linked to non-moral dispositions such as authoritarianism and social dominance, it did not address the possibility that these moral intuitions would also be linked to system justification and underlying epistemic and existential needs to reduce uncertainty and threat. Furthermore, no previous studies (to our knowledge) have considered the possibility that the endorsement of “individualizing” moral intuitions are linked to a qualitatively different kind of motivation, namely empathic motivation.

Although we employ path modelling techniques, we wish to emphasize that we are in no way attempting to test a *causal* model of moral and political judgment [e.g., 62]. For instance, we make no assumptions about the developmental sequence of moral and political attitude acquisition in childhood or adolescence. Instead, we set out to use the theory of political ideology as motivated social cognition as a means of illuminating the questions of how and why seemingly disparate phenomena—such as epistemic and existential needs, “binding” and “individualizing” moral intuitions, system justification, and political ideology—would fit together and to map out the patterns of empirical relations in a theoretically useful and generative manner. Thus, when we use terms such as “path,” “predict,” and “mediate,” we refer solely to *statistical* relations, such as the observation that one variable is associated with another or accounts for (in statistical terms) the covariation between two other variables, without making any philosophical assumptions about causality.

We also wish to clarify our theoretical rationale for ordering the psychological and political variables in the ways we have in the path model for Study 2. As a general rule, we believe that it makes the most sense to begin with variables that are more general or abstract—such as epistemic and existential needs to reduce uncertainty and threat and general empathic motivation—and to use these to predict other somewhat more specific psychological variables that apply, at least potentially, to non-political as well as political domains, such as specific moral intuitions about fairness, harm avoidance, ingroup loyalty, authority, and purity. Next we move on to variables that are more specific but still somewhat general in their application (such as system justification) and then finally to variables that are more concrete and overtly political (such as liberal-conservative orientation). This is the approach we have taken in several other studies in which we demonstrate that system justification mediates (or helps to account for or partially explain) the effects of general psychological variables (such as epistemic, existential, and relational motives) on specific political attitudes and liberal-conservative self-identification [63,64]. Once again, this does not mean that we are proposing that, for instance, moral intuitions “cause” system justification or political ideology. On the contrary, we are persuaded by the research of Hatemi et al. [29], who argue that moral judgments are *post hoc* rationalizations of ideological preferences, and we regard the research program here as consistent with that approach.

## Study 1

### Method

#### Participants

Two hundred and twenty-five NYU students (Mean age = 18.7 years, *SD* = 1.03; 168 females) participated in a mass-testing session for course credit (Spring 2011). They completed measures of epistemic and existential motivation, empathy, system justification, moral intuitions, and political ideology, and provided demographic information.

#### Epistemic motivation

To measure epistemic motivation, we administered the Need for Cognitive Closure and Need for Cognition scales. With respect to the need for cognitive closure, participants indicated their level of agreement with 42 statements [65], such as: “I don’t like situations that are uncertain” (*α* = .85). With respect to the need for cognition, participants indicated their agreement/disagreement with 18 statements [66], such as: “I only think as hard as I have to” (reverse-scored), and “I would prefer complex to simple problems” (*α* = .87). In both cases, participants used a response scale ranging from 1 (*extremely uncharacteristic*) to 5 (*extremely characteristic*). As expected, scores on these two measures were negatively correlated (*r* = -.39, *p* < .01).

#### Existential motivation

We measured existential motivation with three items used in prior research to measure death anxiety [67,68], namely: “I have an intense fear of death”, “It annoys me to hear about death”, and “I would never accept a job in a funeral home.” Participants indicated their level of agreement with these statements on a scale ranging from 1 (*strongly disagree*) to 9 (*strongly agree*). In our sample, reliability was rather low (*α* = .48), which is partially attributable to the small number of items [69].

#### Empathy

To measure empathic sensitivity, we administered the 20-item Basic Empathy Scale [70], which contains items such as, “I can usually realize quickly when a friend is angry” (*α* = .81). Participants indicated their agreement on a scale ranging from 1 (*strongly disagree*) to 5 (*strongly agree*).

#### Moral intuitions

We administered the 20-item Moral Foundations Questionnaire [71] to gauge individual differences in moral intuitions. The first 10 questions addressed the perceived moral relevance of factors such as “Whether or not someone conformed to the traditions of society,” and “Whether or not someone cared for someone weak or vulnerable” ranging from 0 (*not at all relevant*) to 5 (*extremely relevant*). The next 10 items required participants to indicate their agreement or disagreement with statements such as “Respect for authority is something all children need to learn” on a scale ranging from 0 (*strongly disagree*) to 5 (*strongly agree*). These two sets of items were then combined to compute moral intuition scores at the individual level. Reliability for some of the foundations was very low (.49 ≤ *α* ≤ .70), as in previous research [30]. By aggregating them into “binding” and “individualizing” clusters of intuitions, we were able to improve reliability considerable (*α* = .80 and .75, respectively).

#### System justification

To assess individual differences in the tendency to justify the societal status quo, we administered the 17-item Economic System Justification Scale [72]. Sample items include: “Most people who don’t get ahead in our society should not blame the system; they have only themselves to blame,” and “There are many reasons to think that the economic system is unfair” (reverse-coded; α = .79). Participants indicated their agreement on a scale ranging from 1 (*strongly disagree*) to 9 (*strongly agree*).

#### Political ideology

To estimate political ideology we calculated the mean of responses to three items used by [73]: (1) “Where on the following scale of political orientation (from extremely liberal to extremely conservative) would you place yourself (overall, in general)?”; (2) “In terms of social and cultural issues in particular, how liberal or conservative are you?”; and (3) “In terms of economic issues in particular, how liberal or conservative are you?” For each item, participants located themselves on scales ranging from 1 (*extremely liberal*) to 11 (*extremely conservative*; *α* = .79).

## Results and discussion

In an attempt to integrate insights derived from theories of moral foundations and political conservatism as motivated social cognition, we assessed three sets of relations in this research program: (a) the extent to which epistemic and existential motives (as well as empathy) predicted moral intuitions, (b) the relationship between moral intuitions and system justification, and (c) the extent to which moral intuitions and system justification mediate the effects of epistemic and existential motivation on political ideology. Correlations among study variables are listed in Table 1.

We hypothesized that epistemic and existential motives would explain a significant amount of variance in moral intuitions, especially with respect to “binding” intuitions. We also hypothesized that empathic concerns would explain a significant amount of variance with respect to intuitions about fairness and the avoidance of harm. As shown in Table 1, epistemic and existential needs to reduce uncertainty and threat were positively correlated with the endorsement of “binding” (but not “individualizing”) moral intuitions, whereas empathic sensitivity was positively correlated with the endorsement of “individualizing” (but not “binding”) moral intuitions. As in previous research [59,63], epistemic and existential needs to reduce uncertainty and threat were positively correlated with system justification and political conservatism. As expected, system justification and political conservatism were positively associated with the endorsement of “binding” moral concerns and negatively associated with the endorsement of “individualizing” moral concerns.

In a further test of our hypotheses, we simultaneously regressed each of the psychological factors onto ratings of the five types of moral intuitions. First, we observed that epistemic and existential motives (as well as empathy) played a significant role in the valuation of ingroup loyalty. More specifically, the need for cognition was a negative predictor (*b* = -.22, *SE* = .09, *β* = -.17, *p* < .05), whereas need for closure (*b* = .24, *SE* = .12, *β* = .14, *p* < .05) and death anxiety (*b* = .07, *SE* = .03, *β* = .14, *p* < .05) were positive predictors of ingroup loyalty, as was empathy (*b* = .28, *SE* = .13, *β* = .14, *p* < .05).

Second, we observed that epistemic motives drove concerns about deference to authority. Need for cognition (*b* = -.29, *SE* = .09, *β* = -.21, *p* < .05) was a negative predictor, whereas need for closure (*b* = .41, *SE* = .12, *β* = .23, *p* < .01) was a positive predictor of deference to authority. No other factors attained statistical significance.

Third, we observed that epistemic and existential motives played a significant role in the valuation of purity. Need for cognition was a negative predictor (*b* = -.28, *SE* = .11, *β* = -.17, *p* < .05), whereas need for closure (*b* = .40, *SE* = .15, *β* = .18, *p* < .001) and death anxiety (*b* = .15, *SE* = .04, *β* = .25, *p* < .001) were positive predictors of purity concerns. Empathy played no significant role in purity concerns.

Fourth, we observed that concerns about fairness were predicted by empathy (*b* = .47, *SE* = .12, *β* = .26, *p* < .001) and were unrelated to epistemic and existential motives. The same pattern was observed with respect to avoidance of harm. Empathy was the only significant predictor (*b* = .79, *SE* = .13, *β* = .38, *p* < .001)

Next we aggregated ratings of the three “binding” intuitions (ingroup loyalty, authority, and purity) and the two “individualizing” intuitions (fairness, harm avoidance) and conducted two more regression models on these aggregated dependent variables. An even starker picture emerged, in comparison with the one based on separate ratings of each intuition, as in previous research [24,27]. The only statistically significant predictors of binding concerns were epistemic and existential motives. Need for cognition was a negative predictor (*b* = -.26, *SE* = .08, *β* = -.22, *p* < .01), whereas need for closure (*b* = .35, *SE* = .11, *β* = .22, *p* < .01) and death anxiety (*b* = .09, *SE* = .03, *β* = .20, *p* < .01) were positive predictors. Empathy played no role. The full model accounted for 21% of the statistical variability in the valuation of binding concerns. By contrast, the only statistically significant predictor of individualizing concerns was empathy (*b* = .63, *SE* = .11, *β* = .36, *p* < .01). The epistemic and existential motives were unrelated to concerns about fairness and harm avoidance. The model accounted for 16% of the statistical variability in the valuation of individualizing concerns.

Thus, the results of our first study were indeed supportive of our integrative model. Epistemic and existential motives were positively associated with the endorsement of “binding” (but not “individualizing”) foundations, as hypothesized. These findings are consistent with the notion that theories of moral foundations and motivated social cognition are compatible rather than incompatible and that the latter perspective can help to explain *why* conservatives value ingroup loyalty, obedience to authority, and purity in the first place. Contrary to the claims of Haidt and Graham [21], it would appear that epistemic and existential needs do indeed play a meaningful role. We also observed that empathic motivation was associated with the endorsement of “individualizing” (but not “binding”) foundations. These findings are consistent with the notion that liberalism and conservatism are both motivated by psychological concerns, but they are qualitatively different psychological concerns.

## Study 2

**Fig 1:**
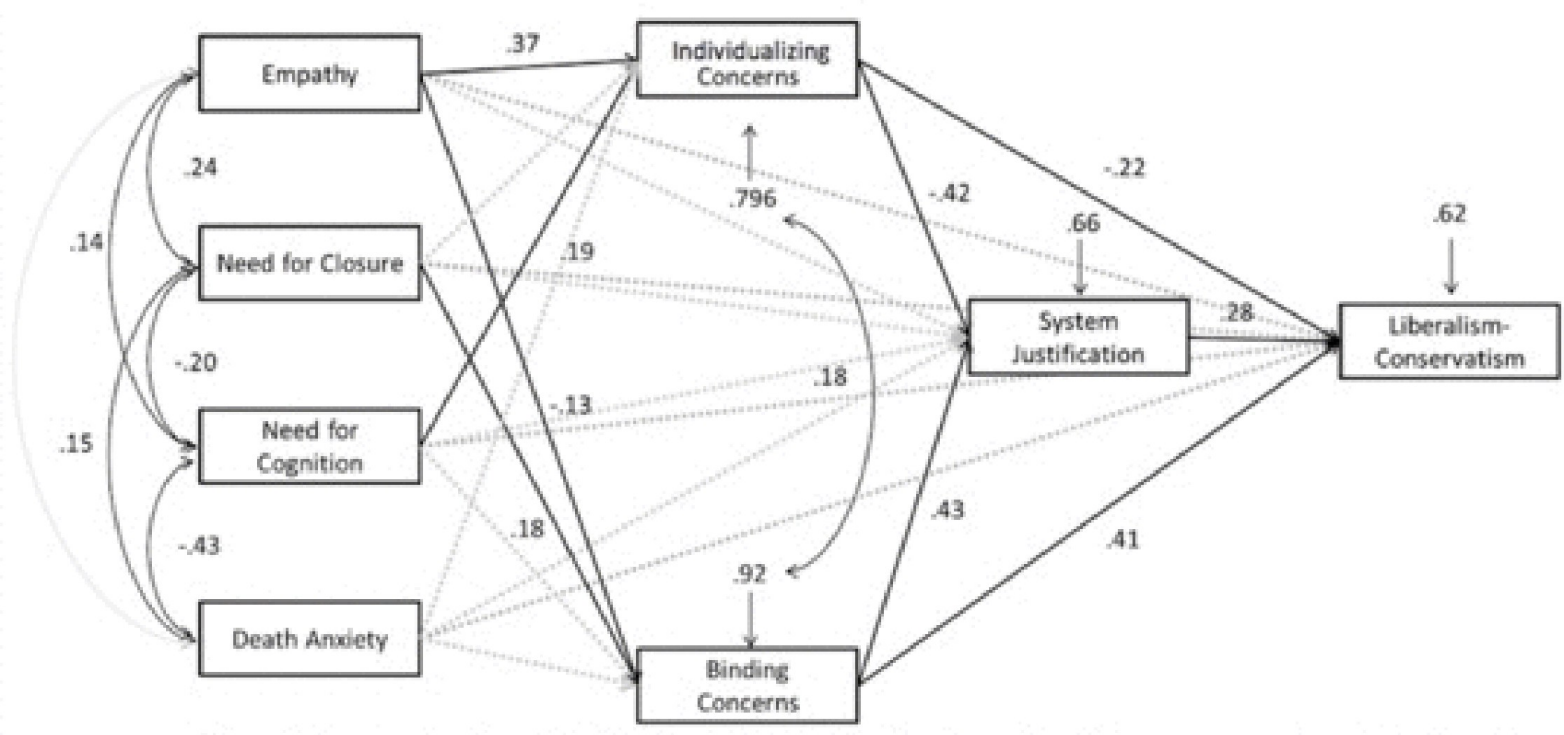
Study 2 path model. Saturated path model of data from Study 2 illustrating the relationships among empathy, epistemic and existential motives, moral foundations, economic system justification, and political orientation. All direct effects were included in the model. Dashed lines indicate insignificant effects.

To develop a broader conceptual framework as a means of integrating theories of moral foundations and ideology as motivated social cognition and to explore the hypothesis that system justification would mediate the effects of moral intuitions on political ideology, we conducted a second study based on a larger and more heterogeneous sample. We also sought to improve upon the reliability of our measurement of death anxiety. Our primary aim, however, was to collect data on a sample that would be suitable to test the path model illustrated in Fig 1 and that would enable us to replicate and extend the findings from our first study.

### Method

#### Participants

Two hundred and seventy-two participants filled out our survey on Amazon’s Mechanical Turk in exchange for $.50 [74], for a detailed discussion of this research platform). We removed 42 participants from further analysis because they either did not pass our attention check question (*n* = 25), responded too quickly (< 4 min) to our survey (*n =* 4), or did not respond to three or more scales (*n* = 15). The remaining two-hundred and forty-nine participants (Mean age = 38.2 years, *SD* = 13.6; 136 females) completed measures of epistemic and existential needs, empathy, system justification, moral intuitions, and political ideology, as well as demographic information.

#### Epistemic motivation

To measure epistemic motivation, we administered the same two scales used in Study 1, namely the Need for Cognitive Closure scale (*α* = .89) and the Need for Cognition scale (*α* = .94).

#### Existential motivation

We tried to address the low reliability score of our measure for existential motivation in study 1 through utilizing a more established death anxiety scale. We administered the 13-item Death Anxiety scale [67,68] containing items such as: “I have an intense fear of death.” Participants indicated their level of agreement with these statements on a scale ranging from 1 (*strongly disagree*) to 7 (*strongly agree*). Reliability of the scale was high this time (*α* = .94).

#### Empathy

To measure empathic concern, we administered the 20-item Basic Empathy Scale as used also in Study 1 (*α* = .89).

#### Moral intuitions

In Study 2, we administered the updated 30-item Moral Foundations Questionnaire [75] to help improve the low reliabilities observed in Study 1 using the older 20-item questionnaire. The first 15 questions addressed the perceived moral relevance of factors such as “Whether or not someone conformed to the traditions of society,” and “Whether or not someone cared for someone weak or vulnerable” ranging from 1 (*not at all relevant*) to 6 (*extremely relevant*). The next 15 items required participants to indicate their agreement with statements such as “Respect for authority is something all children need to learn” on a scale ranging from 1 (*strongly disagree*) to 6 (*strongly agree*). These two sets of items are then combined to compute moral intuition scores at the individual level. For our MTurk sample, subscale reliabilities were all acceptable: Avoidance of Harm (*α* = .70), Fairness (*α =* .68), Ingroup Loyalty (*α* = .69), Obedience to Authority (*α* = .74), and Purity (*α* = .83); by aggregating them into “individualizing” and “binding” intuitions, we were able to improve reliabilities further (*α* = .81 and .89, respectively).

#### System justification

In Study 2, we administered the same 17-item Economic System Justification Scale as in Study 1. Reliability was once again satisfactory (*α* = .87).

#### Political ideology

The same three questions were used to assess participants’ political ideology. Reliability was higher in this sample (*α* = .94).

## Results and discussion

As shown in Table 2, epistemic and existential needs to reduce uncertainty and threat were again positively correlated with the endorsement of “binding” moral intuitions. Somewhat surprisingly, need for cognitive closure and need for cognition—despite being negatively correlated with one another, as in previous research—were both positively associated with the endorsement of “individualizing” moral intuitions in this study. As in Study 1, empathic sensitivity was positively correlated with the endorsement of “individualizing” (but not “binding”) moral concerns. Once again, system justification and political conservatism were positively associated with the endorsement of “binding” moral concerns and negatively associated with the endorsement of “individualizing” moral concerns.

**Table 2:** Descriptive statistics including intercorrelations of study variables (Study 2, *N* = 249 Amazon mTurk).

		*M*	*SD*	1	2	3	4	5	6	7	8	9	10	11	12
**Motivation Variables**	(1) Basic Empathy	3.57	0.53	-											
	(2) Need for Closure	3.96	0.53	.24**	-										
	(3) Need for Cognition	3.38	0.84	.14*	-.20**	-									
	(4) Death Anxiety	3.73	1.45	-.09	.15*	-.43**	-								
**Moral Intuition Variables**	(5) Avoidance of Harm	4.70	0.87	.40**	.13*	.13*	-.03	-							
	(6) Fairness Concerns	4.81	0.86	.35**	.13*	.26**	-.11	.63**	-						
	(7) Ingroup Loyalty	3.78	0.94	-.04	.07	-.03	.14*	.16**	.15*	-					
	(8) Obedience to Authority	3.73	1.00	-.04	.23**	-.22**	.07	.02	.01	.61**	-				
	(9) Purity Concerns	3.67	1.22	-.16**	.15*	-.23**	.19**	.07	.05	.47**	.69**	-			
	(10) Humanizing Concerns	4.75	0.78	.42**	.14*	.21**	-.07	.91**	.90**	.17**	.02	.07	-		
	(11) Binding Concerns	3.72	0.90	-.10	.18**	-.19**	.16*	.10	.08	.79**	.90**	.87**	.10	-	
**Ideology**	(12) Economic SJ	4.52	1.32	-.24**	.07	-.16*	.13*	-.36**	-.33**	.31**	.42**	.31**	-.38**	.40**	-
	(13) Liberalism-Conservatism	4.51	2.24	-.12	.10	-.09	.02	-.23**	-.21**	.33**	.52**	.38**	-.24**	.48**	.50**

Note. SJ = Systemic Justification

**. Correlation is significant at the .01 level (2-tailed).

*. Correlation is significant at the .05 level (2-tailed).

We also performed a regression analysis that paralleled the analysis from the previous study. First, we observed that while existential motivation played a significant role in the valuation of ingroup loyalty (as in Study 1), neither epistemic motivation nor empathy played a significant role. Death anxiety was again a positive predictor of ingroup loyalty (*b* = .10, *SE* = .05, *β* = .14, *p* < .05), but no other factor attained statistical significance. Second, we observed that (as in Study 1) epistemic motivation drove concerns about obedience to authority. Need for cognition (*b* = -.23, *SE* = .08, *β* = -.19, *p* < .01) was a negative predictor, whereas need for closure (*b* = .41, *SE* = .12, *β* = .22, *p* = .001) was a positive predictor of obedience to authority. No other factors attained statistical significance.

Third, we observed that epistemic motivation played a significant role in the valuation of purity (as in Study 1). Need for closure was a positive predictor (*b* = .35, *SE* = .15, *β* = .15, *p* < .05), whereas need for cognition was a negative predictor (*b* = -.20, *SE* = .10, *β* = -.14, *p* = .05) of purity concerns. Empathy was also a negative predictor of purity concerns (*b* = -.40, *SE* = .15, *β* = -.17, *p* < .01). Death anxiety, however, played no significant role. Fourth, we observed that concerns about fairness were once again predicted by empathy (*b* = .48, *SE* = .10, *β* = .30, *p* < .001) as well as need for cognition (*b* = .25, *SE* = .07, *β* = .24, *p* < .001)—but not need for closure. As in Study 1, there was no association between existential motivation and fairness concerns. Fifth and finally, avoidance of harm was predicted by empathy (*b* = .62, *SE* = .10, *β* = .38, *p* < .001), but it was unrelated to epistemic and existential motivation (as in Study 1).

Calculating the same regression model for the aggregated individualizing and binding foundations, we obtained slightly different results than in Study 1. In terms of the binding foundations, need for closure (*b* = .30, *SE* = .11, *β* = .18, *p* < .01) was again a positive predictor, whereas empathy was a marginally significant negative predictor (*b* = -.21, *SE* = .11, *β* = -.13, *p* = .055). In this case, existential motivation played no significant role. The model accounted for 8% of the statistical variability in the valuation of binding concerns. By comparison, the statistically significant predictors of individualizing concerns were empathy (*b* = .54, *SE* = .09, *β* = .37, *p* < .001) and need for cognition (*b* = .18, *SE* = .06, *β* = .19, *p* < .01). Existential motivation was unrelated to these concerns. The model accounted for 20% of the statistical variability in the valuation of individualizing concerns.

To develop a broader conceptual framework that integrates theories of moral foundations and ideology as motivated social cognition and to investigate the hypothesis that system justification would mediate the effects of moral foundations on political orientation, we compared and contrasted two path models using MPlus 7 [76]. We assessed a model in which (a) empathy would predict individualizing concerns, whereas epistemic and existential motives would predict binding concerns, (b) the two types of moral foundations would exert opposite effects on system justification, and (c) system justification would mediate the effects of moral foundations on political orientation (see Fig 1).

We first built the saturated model illustrated in Fig 1. Such a saturated, manifest variable model has *χ*^2^, RMSEA, and SRMR values of 0, and CFI and TLI values of 1. The model yielded positive associations between empathy and endorsement of individualizing concerns, *b* = .54, *SE* = .10, *β* = .37, *p* < .001. Need for cognition was also positively associated with individualizing concerns, *b* = .18, *SE* = .07, *β* = .19, *p* < .01. Existential motivation was unrelated to individualizing concerns (*b* = .13, *SE* = .11, *β* = .09, *p* = .25; and *b* = .02, *SE* = .04, *β* = .03, *p* = .64 respectively). Need for closure was positively associated with the endorsement of binding concerns (*b* = .30, *SE* = .11, *β* = .18, *p* < .01), and there was a marginally significant negative association between empathy and endorsement of binding concerns (*b* = -.21, *SE* = .11, *β* = -.13, *p* = .057).

As hypothesized, system justification was negatively associated with individualizing concerns (*b* = -.71, *SE* = .10, *β* = -.42, *p* < .001) and positively associated with binding concerns (*b* = .63, *SE* = .08, *β* = .43, *p* < .001). The individualizing and binding concerns mediated the relations between empathy, need for closure, need for cognition, and death anxiety, on one hand, and system justification, on the other. In addition, political conservatism was positively associated with system justification (*b* = .48, *SE* = .12, *β* = .28, *p* < .001) and with the endorsement of binding concerns (*b* = 1.02, *SE* = .16, *β* = .41, *p* < .001). Conservatism was negatively associated with the endorsement of individualizing concerns (*b* = -.62, *SE* = .18, *β* = -22, *p* < .001). Empathy, existential, and epistemic motivations explained 20.4% of the variance in endorsement of individualizing concerns and 7.8% of the variance in the endorsement of binding concerns. The model explained 34.2% of the variance in system justification and 37.9% of the variance in political orientation.

We then estimated individual indirect paths using the bootstrapping technique [77,78]. We obtained 95% confidence intervals using 5,000 samples. An indirect effect is considered significant if the unstandardized 95% confidence interval around the estimate does not contain zero. We observed that individualizing concerns mediated the effects of empathy and need for cognition on system justification ({-.55, -.25} and {-0.211, -.052}, respectively). Binding concerns mediated the effects of empathy and need for closure on system justification ({-0.271, -.025} and {.069, .311}, respectively). Neither type of moral concern was found to mediate the effect of death anxiety on system justification. These results indicate that moral concerns significantly mediated the effects of empathy and epistemic motivation (but not existential motivation) on system justification.

In terms of the indirect paths from psychological motives to moral concerns to political ideology, we observed that individualizing concerns mediated the effect of need for cognition on ideology {-0.23, -.04}. Binding concerns mediated the effects of empathy {-0.43, -0.05} and need for closure {0.12, .52} on ideology. With respect to the full indirect paths, the following were statistically significant: (Empathy → Individualizing concerns → System Justification → Political Ideology): {-0.31, -0.10}, (Empathy → Binding concerns → System Justification → Political Ideology): {-0.15, -.02}, (Need for Closure → Binding concerns → System Justification → Political Ideology): {0.03, .18}, (Need for Cognition→ Individualizing concerns → System Justification → Political Ideology): {-.11, -.03}, and (Need for Cognition→ Binding concerns → System Justification → Political Ideology): {-0.09, -0.002}.

The results of Study 2 provide additional evidence in support of our integrative model, especially with respect to the path model illustrated in Fig 1. In terms of epistemic motives, the need for cognitive closure was positively associated with the endorsement of “binding” but not “individualizing” foundations, whereas the need for cognition was positively associated with the endorsement of “individualizing” but not “binding” foundations. Furthermore, empathy was positively associated with “individualizing” concerns, but it was negatively associated with “binding” concerns. Both of these dissociations would be well worth exploring in future research. Finally, system justification mediated the (opposite) effects of individualizing and binding concerns on political ideology. These findings suggest that liberalism and conservatism are indeed associated with qualitatively different psychological concerns. They are also consistent with the notion that theories of moral foundations and motivated social cognition are compatible rather than incompatible—and that the latter perspective may be useful for explaining conservatives’ prioritization of ingroup loyalty, obedience to authority, and purity in the first place. Thus, our findings buttress the theoretical argument advanced by Hatemi et al. [29], namely that moral intuitions are themselves the product of motivated social cognition.

## General discussion

There are at least two ways of making sense of the present findings in light of the broader research literature on moral foundations theory. One possibility is that moral intuitions represent something that is relatively stable and coherent about the individual’s psychological make-up—something that is influenced by underlying epistemic and existential needs to reduce uncertainty and threat. For instance, concerns about maintaining intergroup boundaries, obeying authority, and enforcing purity sanctions in the moral domain may, at least in part, reflect underlying individual differences in the desire for an orderly and predictable world in which nothing is left to chance. This could explain, for instance, why the need for cognitive closure is positively associated with the endorsement of “binding foundations.” This possibility is consistent with previous studies conducted by Federico and colleagues [36] but seemingly inconsistent with the analysis of Hatemi et al. [29], which suggested that moral intuitions are little more than *post hoc* rationalizations of left-right ideological preferences.

Another possibility is that moral foundations are merely epiphenomenal reflections of more stable ideological proclivities that are themselves tied to the individual’s psychological make-up. The idea here is that individual differences in the strength of epistemic and existential needs to reduce uncertainty and threat give rise to “liberal” and “conservative” personalities and corresponding ideological preferences [13,14], and these, in turn, lead people to embrace certain moral intuitions and judgments. This view, which is more consistent with the results of research by Hatemi and colleagues (29), implies a much stronger critique of moral foundations theory. This is because it undermines the assumption, which we regard as dubious for a number of reasons alluded to in the introductory section of this article, that conservatives and those who are high in authoritarianism and social dominance are primarily motivated by authentic moral concerns rather than other psychological motives that are best regarded as amoral if not immoral [23].

## Concluding remarks

Taken in conjunction, the results presented here lead to several conclusions that should be of relevance to social scientists who study morality, social justice, and political ideology. First, we observe that so-called “binding” moral concerns pertaining to ingroup loyalty, authority, and purity are psychologically linked to epistemic and, to a lesser extent, existential motives to reduce uncertainty and threat. Second, so-called “individualizing” concerns for fairness and avoidance of harm are not linked to these same motives. Rather, they seem to be driven largely by empathic sensitivity. Third, it would appear that theories of moral foundations and motivated social cognition are in some sense compatible, as suggested by Van Leeuween and Park [45], rather than incompatible, as suggested by Haidt and Graham [21] and Haidt [22]. That is, the motivational basis of conservative preferences for “binding” intuitions seems to be no different than the motivational basis for many other conservative preferences, including system justification and the epistemic and existential motives that are presumed to underlie system justification [13,14,59,63].

Fourth, moral foundations theory may be useful for fleshing out the picture of conservatism initiated by the theory of political ideology as motivated social cognition [13,14], insofar as “binding” and “individualizing” concerns were indeed found to mediate the effects of epistemic motivation on system justification. Fifth, we have provided additional evidence that the endorsement of “individualizing” concerns is more closely linked to prosocial orientations—such as empathic sensitivity—than is the endorsement of “binding” concerns. In fact, empathic sensitivity was *negatively* associated with the endorsement of “binding” concerns, consistent with prior evidence linking “binding” concerns to prejudice, outgroup hostility, and other antisocial outcomes. Sixth, our findings echo the conclusions of Hatemi et al. [29], who argued that—rather than building ideological edifices on stable moral principles or “foundations”—people tend to “use morality to justify their preexisting political values at least as much, if not more than, using morality to inform them” (p. 3).

Far from indicating that ideological differences between liberals and conservatives are attributable to exclusively “moral” concerns [21]—or to “transparent” factors rather than “subterranean motives” [20]—our findings are consistent with a century or more of theorizing about the motivational underpinnings of political attitudes. When people prioritize ingroup loyalty, obedience to authority, and the enforcement of purity sanctions, it seems quite likely that they are, among other things, expressing underlying desires for certainty, predictability, order, control, safety, security, and reassurance. This is not necessarily the case for people who prioritize fairness and the avoidance of harm; their values appear to be more closely linked to empathic concerns. Thus, when Haidt and Graham [21] observe that liberals are more committed to social justice than conservatives, they may in fact be saying something much deeper and more profound than they realize about the nature of individual differences in “moral” motivation.

To underscore the role of ideological polarization when it comes to ideals of social justice, Rothmund and colleagues [79] juxtaposed the perspectives of Che Guevara on the left and Friedrich von Hayek on the right. Whereas Guevara declared that, “If you tremble with indignation at every injustice then you are a comrade of mine,” von Hayek warned that the “prevailing belief in ‘social justice’ is at present probably the gravest threat to most other values of a free civilization.” The challenge, and indeed the promise, of social, personality, and political psychology, it seems to us, is to determine how and why the same stimulus—in this case, social justice—is so appealing to some and so threatening to others.

## Supporting information

S1 Data (ZIP)
